# Nontranscriptional activation of PI3K/Akt signaling mediates hypotensive effect following activation of estrogen receptor β in the rostral ventrolateral medulla of rats

**DOI:** 10.1186/1423-0127-19-76

**Published:** 2012-08-16

**Authors:** Kay LH Wu, Chen-Hsiu Chen, Cheng-Dean Shih

**Affiliations:** 1 Center for Translational Research in Biomedical Sciences, Kaohsiung Chang-Gung Memorial Hospital, Kaohsiung, 83301, Taiwan; 2 Department of Anesthesiology, Kaohsiung Veterans General Hospital, Kaohsiung, 81346, Taiwan; 3 School of Medicine, National Yang-Ming University, Taipei, 11221, Taiwan; 4 Department of Pharmacy & Graduate Institute of Pharmaceutical Technology, Tajen University, 20 Weishin Road, Yanpu Township, Pingtung County, 90741, Taiwan

**Keywords:** 17β-estradiol, Estrogen receptor, Rostral ventrolateral medulla, Systemic arterial pressure, PI3K/Akt signaling pathway

## Abstract

**Background:**

Estrogen acts on the rostral ventrolateral medulla (RVLM), where sympathetic premotor neurons are located, to elicit vasodepressor effects via an estrogen receptor (ER)β-dependent mechanism. We investigated in the present study nontranscriptional mechanism on cardiovascular effects following activation of ERβ in the RVLM, and delineated the involvement of phosphatidylinositol 3-kinase (PI3K)/serine/threonine kinase (Akt) signaling pathway in the effects.

**Methods:**

In male Sprague–Dawley rats maintained under propofol anesthesia, changes in arterial pressure, heart rate and sympathetic neurogenic vasomotor tone were examined after microinjection bilaterally into RVLM of 17β-estradiol (E2β) or a selective ERα or ERβ agonist. Involvement of ER subtypes and PI3K/Akt signaling pathway in the induced cardiovascular effects were studied using pharmacological tools of antagonists or inhibitors, gene manipulation with antisense oligonucleotide (ASON) or adenovirus-mediated gene transfection.

**Results:**

Similar to E2β (1 pmol), microinjection of ERβ agonist, diarylpropionitrile (DPN, 1, 2 or 5 pmol), into bilateral RVLM evoked dose-dependent hypotension and reduction in sympathetic neurogenic vasomotor tone. These vasodepressive effects of DPN (2 pmol) were inhibited by ERβ antagonist, R,R-tetrahydrochrysene (50 pmol), ASON against ERβ mRNA (250 pmol), PI3K inhibitor LY294002 (5 pmol), or Akt inhibitor (250 pmol), but not by ERα inhibitor, methyl-piperidino-pyrazole (1 nmol), or transcription inhibitor, actinomycin D (5 or 10 nmol). Gene transfer by microinjection into bilateral RVLM of adenovirus encoding phosphatase and tensin homologues deleted on chromosome 10 (5 × 10^8^ pfu) reversed the vasodepressive effects of DPN.

**Conclusions:**

Our results indicate that vasodepressive effects following activation of ERβ in RVLM are mediated by nongenomic activation of PI3K/Akt signaling pathway. This study provides new insight in the intracellular signaling cascades involved in central vasodepressive functions of estrogen.

## Background

The primary female sex steroid, estrogen has long been recognized as an important hormone in a wide variety of physiological processes, including the development, growth, metabolism, reproduction, cardiovascular and sexual function in both humans and animals [1]. Most of these biological actions appear to be mediated through the estrogen receptor (ER) subtypes, ERα and ERβ, a member of the nuclear receptor superfamily that function as ligand-activated transcriptional factors [2,3]. Upon binding to its ligand, ER forms homodimers or heterodimers between the subtypes, and bind to a specific DNA sequence located in the promoter region of target genes where it regulates gene expression through direct interactions with DNA or other transcriptional machinery proteins [2,3].

It is now recognized that not all biological effects of estrogen are mediated by this well-established transcriptional mode of action. Recent studies have shown that a subpopulation of the traditional nuclear ERs is localized at the cell membrane and they mediate many of the rapid signaling actions of estrogen via nontranscriptional mechanisms [3-7]. In the heart and peripheral blood vessels, nongenomic mechanisms underlie estrogen-induced rapid arterial vasodilation [5,6], inhibition of atherosclerotic lesions [5,8], cardioprotective effect following trauma-hemorrhage [7], and amelioration of ischemia/reperfusion-induced myocardial injury [9]. These nongenomic actions of estrogen are thought to be mediated via activation of the phosphatidylinositol 3-kinase (PI3K) [5-7] and serine/threonine kinase Akt (Akt) [5,7] signaling cascades following direct activation by the hormone of the cellular membrane rather than intracellular receptors [4,5,7,10].

Besides their well-known peripheral roles in cardiovascular functions, accumulating evidence indicates that estrogen in the brain also participates in the regulation of cardiovascular functions [11-18]. Central administration of estrogen effectively augments sympathetic nerve activity resulting in the increase in systemic arterial pressure (SAP) in female rats [11]. In estrogen-replaced ovariectomized female rats [16], but not in male rats [15], peripheral injection of estrogen decreases baseline sympathetic outflow and SAP that are antagonized by central injection of the ER antagonist [15,16]. In addition, immunohistochemical [19,20] and *in situ* hybridization studies [19,20] have demonstrated the distribution of both ER mRNA and protein in neurons of the rostral ventrolateral medulla (RVLM), where the sympathetic premotor neurons are located [21]. These anatomical findings suggest this nucleus as a candidate substrate to subserve the central cardiovascular regulatory actions of estrogen. In this regard, microinjection of estrogen into the RVLM causes a rapid suppression in autonomic vasomotor tone and SAP [13,14,17]. Moreover, our recent study [17] reported that activation of ERβ in the RVLM mediates the reduction in autonomic functions produced by estrogen. Contributions of nongenomic mechanisms and its downstream signaling pathways underlying the central cardiovascular effects of ERβ activation in the RVLM, however, are unexplored. The present study was undertaken to assess the hypothesis that ERβ activation in the RVLM participates in neural regulation of cardiovascular functions via nongenomic mechanisms that entail activation of PI3K/Akt signaling pathways. Our results demonstrated that estrogen in the RVLM evoked vasodepressive effects through activation of ERβ, but not ERα receptors, and nongenomic activation of PI3K/Akt signaling pathway.

## Methods

### Animals

All animal care and experimental protocols were approved by Institutional Animal Care and Use Committee of Tajen University, and were in accordance with the *Guide for the Care and Use of Laboratory Animals* published by the U.S. National Institutes of Health (NIH Publication No. 85–23, revised 1996). Adult male Sprague–Dawley rats weighing 250–300 g (n = 142) were purchased from the Experimental Animal Center of the National Science Council and BioLASCO, Taiwan, Republic of China. They were housed under conditions of constant temperature (23 ± 0.5 °C) and humidity (50 ± 5 %) with a standard 12 h light–dark cycle (08:00–20:00) and unrestricted access to standard laboratory rat chow (Purina) and tap water. All animals were allowed to acclimatize for at least 7 days before experimental manipulations. All efforts were made to reduce the numbers of animals used and to minimize animal suffering at each stage of the experiment.

### General animal preparation

The preparatory surgery that included intubation of the trachea and cannulation of the femoral artery and both femoral veins was performed under an induction dose of pentobarbital sodium (50 mg/kg, i.p.) [17,22]. During recording session, the anesthesia was maintained by continuous intravenous infusion of propofol (Zeneca Pharmaceuticals, Macclesfield, UK) at 20–25 mg/kg/h, which provided satisfactory anesthetic maintenance while preserving the capacity of neural control of cardiovascular functions [23]. Pulsatile and mean SAP (MSAP), as well as heart rate (HR), was continuously displayed on a polygraph (Gould RS3400, Valley View, OH, USA) via a pressure transducer (BD P23XL, Franklin Lakes, NJ, USA). Animals were ventilated mechanically by the use of a small rodent ventilator (Harvard 683, South Natik, MA, USA) to maintain an end-tidal CO_2_ within 4.0-4.5 %, as monitored by a capnograph (Datex Normocap, Helsinki, Finland). This procedure was conducted to minimize possible confounding cardiovascular changes secondary to respiratory perturbation. The head of the animal was thereafter fixed to a stereotaxic head holder (Kopf 1430, Tujunga, CA, USA), and the rest of the body was placed on a thermostatically controlled pad to maintain rectal temperature of 37 ± 0.5 °C. All data were collected from animals with a steady baseline MSAP above 90 mmHg at the beginning of the recording period.

### Recording and power spectral analysis of SAP signals

The waveform signals of SAP recorded from the femoral artery were simultaneously subject to online power spectral analysis as detailed previously [23-25]. The SAP signals were resampled at 256 Hz by an eight-point averaging algorithm, and analyzed was truncated into 16-s (2,048 points) time segments with 50 % overlap. A Hamming window in the time domain was used to decline the leakage effect [24,26]. Power spectral density of different frequency components of SAP signals was computed using a fast Fourier transform. We were particularly interested in the very low-frequency (0–0.25 Hz) and low-frequency (0.25-0.8 Hz) components of the SAP spectrum. These frequency components of SAP signals were reported to take origin from the RVLM [25] and their power density reflect the prevailing sympathetic neurogenic vasomotor tone [17,22,23,25,27]. The power densities of these two spectral components were displayed during the experiment, alongside SAP, MSAP and HR, in an online and real-time manner.

### Microinjection of test agents into the RVLM

Similar to the procedures described previously [17,27], microinjection bilaterally of test agents into the functionally identified RVLM sites was performed stereotaxically and sequentially with a glass micropipette (external tip diameter: 50–80 μm) connected to a 0.5-μL Hamilton microsyringe (Reno, NV, USA). The stereotaxic coordinates for the RVLM were 4.5 to 5.0 mm posterior to lambda, 1.8 to 2.1 mm lateral to midline, and 8.0 to 8.5 mm below the dorsal surface of the cerebellum. These coordinates were selected to cover the ventrolateral medulla in which both ER mRNA [19] and protein [20,28] are distributed, and where functionally identified sympathetic premotor neurons are located [21]. At the beginning of each experiment, the functional location of RVLM neurons on either side was established by monitoring a transient pressor response (15–25 mmHg) after microinjection of L-glutamate (1 nmol, Sigma–Aldrich). Subsequent microinjections of test agents were delivered to the identified pressor loci. The test agents used in this study included 17β-estradiol-3-sulphate sodium (E2β; Sigma–Aldrich, St. Louis, MO, USA); a selective ERα agonist, 1,3,5-*tris*(4-hydroxyphenyl)-4-propyl-1 H-pyrazole (PPT; Tocris Cookson Inc., Bristol, UK); a selective ERβ agonist, diarylpropionitrile (DPN; Tocris Cookson); a nonspecific ER antagonist, ICI 182780 (Tocris Cookson); a selective ERα antagonist, methyl-piperidino-pyrazole (MPP; Tocris Cookson); a selective ERβ antagonist, R,R-tetrahydrochrysene (R,R-THC; Tocris Cookson); a transcription inhibitor, actinomycin D (AMD; Tocris Cookson); a PI3K inhibitor, LY294002 (Calbiochem, San Diego, CA, USA); an Akt inhibitor (Calbiochem); an antisense oligonucleotide (ASON) against the rat ERα or ERβ mRNA (Quality Systems, Taipei, Taiwan) or the scrambled (SCR) ERα or ERβ oligonucleotide (Quality Systems). For ERα mRNA, the ASON sequence was 5’-CATGGTCATGGTCAG-3’ and the SCR sequence was 5’-ATCGTGGATCGTGAC-3’ [29]. For ERβ mRNA, the ASON sequence was 5’-GAATGTCATAGCTGA-3’ and the SCR sequence was 5’-AAGGTTATCGCAAGT-3’ [29]. A total volume of 50 nl was microinjected to each side of RVLM. The dose of test agents and treatment regimen were adopted from our preliminary experiments and previous studies [17,30] that used the same test agents for the same purpose as in this study. The dose of each antagonist or inhibitor used in this study has been shown in our pilot studies to significantly inhibit cardiovascular responses induced by its specific ligand or enzyme. All test agents were freshly dissolved in artificial cerebrospinal fluid (aCSF) at pH 7.4, except for ICI 182780, AMD and LY294002, which used 5 % dimethyl sulfoxide (DMSO) as the solvent. The composition of aCSF was (in mM): NaCl 117, NaHCO_3_ 25, glucose 11, KCl 4.7, CaCl_2_ 2.5, MgCl_2_ 1.2, and NaH_2_PO_4_ 1.2. Control experiments showed that these vehicles had no significant effect on baseline MSAP or HR during the 120 min observation period. To avoid confounding effects of drug interactions, each animal received only one treatment of synthetic estrogen, selective ERα, ERβ agonist or vehicle, given alone or in combination with one test agent. Pretreatment with microinjection into the bilateral RVLM of AMD, or ERα or ERβ ASON or SCR, were carried out 1 h or 24 h respectively, prior to DPN application.

### Construction and purification of recombinant AdPTEN

To construct adenovirus encoding phosphatase and tensin homologues deleted on chromosome 10 (AdPTEN) or green fluorescence protein (AdGFP), human PTEN complementary DNA or GFP was subcloned into the adenovirus transfer vector pCA13 (Microbix, Toronto, Ontario, Canada) to yield the pCA13-PTEN or pCA13-GFP fusion protein. Subsequently, recombinant adenovirus was generated by cotransfection of pCA13-PTEN or pCA13-GFP with the pJM17 vector (Microbix, Toronto, Ontario, Canada), a plasmid containing the adenoviral genome, into 293 cells [27]. The adenoviruses were purified by CsCl ultracentrifugation and desalted by G-25 gel-filtration chromatography. The titers of the adenoviruses were determined by measuring optical density at 260 nm and plaque-forming assays using 293 cells. AdGFP served as the control viruses [27].

### Adenovirus-mediated PTEN gene transfer into the RVLM

The gene transfer of PTEN into the RVLM was performed by microinjection bilaterally into the nucleus of AdGFP or AdPTEN 7 days prior to DPN administration. An adenoviral suspension containing 5 × 10^8^ plaque-forming units (pfu)/100 nl was administered into each injection site over 10–15 min using a glass micropipette [27]. The coordinates used are the same for microinjection of the test agents. Following the microinjection, the muscle layers covering the fourth ventricle were sutured. Body temperature was maintained at 37 °C with heating pads until the animals had recovered from surgery. The rats were allowed to recover in their home cages with free access to food and water. Only animals that showed progressive weight gain after the gene transfer were used in subsequent experiments.

### Protein extraction and Western blot analysis

For quantitative analysis of PTEN protein expression in the RVLM, the ventrolateral medulla was rapidly removed and placed on dry ice. Tissues on both sides at the level of RVLM (0.5 to 1.5 mm rostral to the obex) that contained the injection sites were collected, and the same medullary samples thus obtained from 4–6 rats under the same experimental condition were pooled and stored at −80 °C until further analysis. Western blot analysis [27,31,32] was carried out on protein extraction from the RVLM for PTEN or β-tubulin. The primary antiserum used included rabbit polyclonal antiserum against PTEN (1:1000; Upstate Biotechnology, Lake Placid, NY, USA) or β-tubulin (1:10000; Sigma–Aldrich). This was followed by incubation with horseradish peroxidase-conjugated goat anti-rabbit IgG (1:10000; Jackson Immunoresearch Laboratories, West Grove, PA, USA). Specific antibody–antigen complex was detected using an enhanced chemiluminescence Western blot detection system (Perkin–Elmer Life Sciences, Boston, MA, USA). The amount of detected protein was quantified by Photo-Print Plus software (ETS Vilber-Lourmat, France) and was expressed as fold change relative to basal PTEN protein level. β-tubulin served as an internal control to demonstrate equal loading of the proteins.

### Immunohistochemical staining

The procedures of immunohistochemical staining were described previously [33]. At day 7 after the gene transfer, rats were deeply anesthetized with pentobarbital sodium (100 mg/kg, i.p.) and perfused transcardially with warm heparinized saline, followed by ice-cold 4 % paraformaldehyde in 0.1 M PBS (pH 7.4). The brain stem was rapidly removed and post-fixed in the latter solution overnight at 4 °C. 35-μm coronal sections of the rostral medulla oblongata were cut using a cryostat. After pre-absorption in gelatin (0.375 %), normal horse serum (3 %) and triton-X 100 (0.2 %) in PBS, the sections were incubated with a rabbit polyclonal antibody against PTEN (1:1000; Wako). After incubation in biotinylated horse anti-mouse IgG (1:200; Jackson ImmunoResearch), the sections were rinsed in PBS and incubated with AB complexes (Vectastain ABC elite kit, Vector Laboratories, Burlingame, CA). This was followed by incubation with a 3,3’-diaminobenzidine substrate kit f (Vector Laboratories). Sections were rinsed in PBS and dehydrated by passing through graded series of ethanol and xylene. Sections were mounted and observed under a light microscope (BX53, Olympus optical, Tokyo, Japan).

### Brain histology

At the conclusion of each experiment, the animal was killed by an overdose of pentobarbital sodium (100 mg/kg, i.p.), and the brain stem was removed from animals and fixed in 30 % sucrose in 10 % formaldehyde–saline solution for at least 72 h. Histological verification of the location of microinjection sites was carried out on frozen 25-μm sections stained with 1 % Neutral red (Sigma–Aldrich).

### Statistical analysis

All values are expressed as mean ± SEM. For functional experiments, the time course of the effects of various treatments on MSAP, HR or power density of vasomotor components of SAP spectrum were assessed using two-way analysis of variance (ANOVA) with repeated measures for group difference. For biochemical experiments, differences between treatment groups were assessed using one-way ANOVA. This was followed by the Scheffé multiple-range test for *post hoc* assessment of individual means. The maximal changes in the hemodynamic parameters were evaluated with Student’s *t*-test. *P* < 0.05 was considered statistically significant.

## Results

### Cardiovascular effects following microinjection bilaterally into the RVLM of E2β, ERα or ERβ agonist

**Figure 1:**
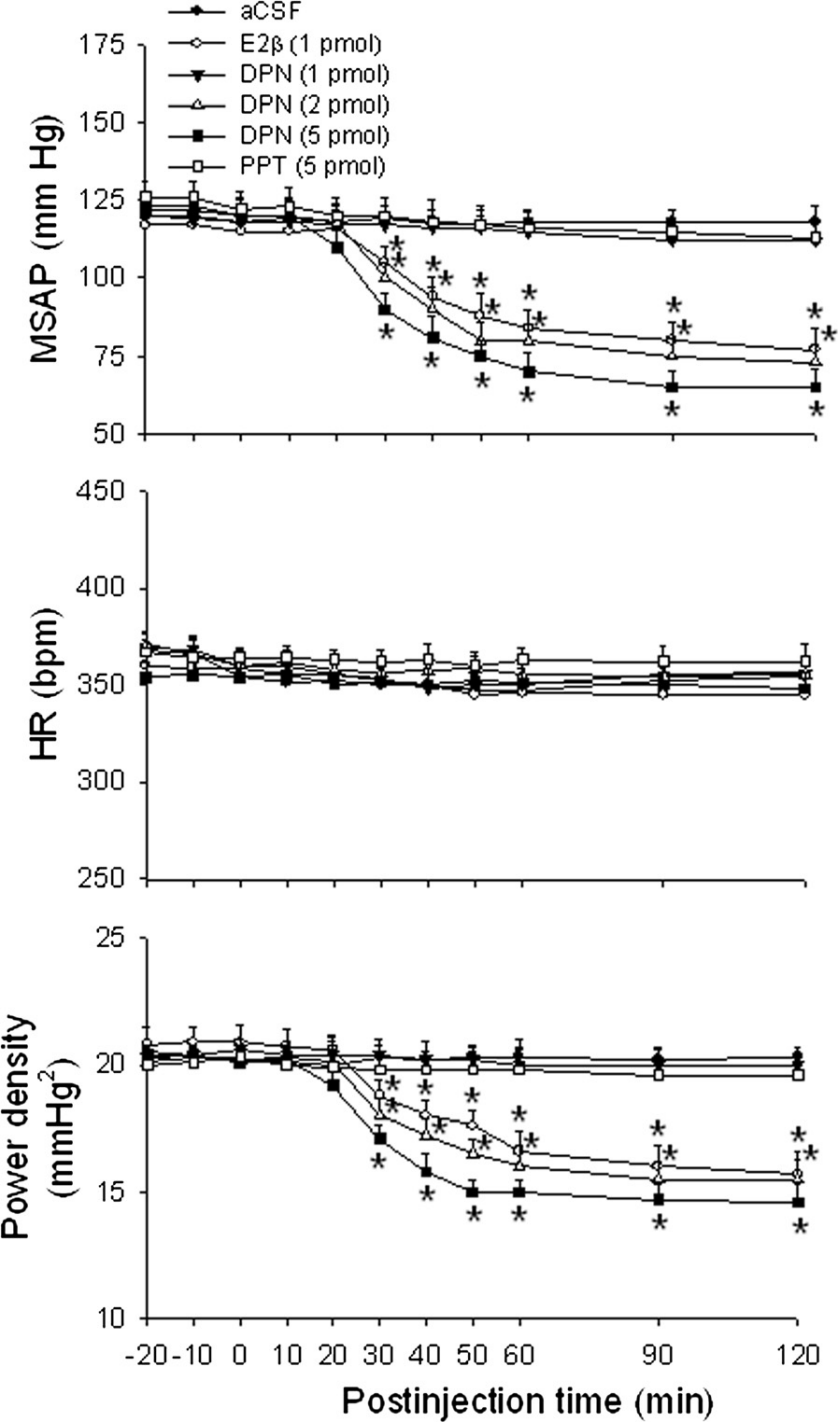
**Cardiovascular effects following microinjection bilaterally into the RVLM of E2β, ERα or ERβ agonist.** Time-course of the changes in mean systemic arterial pressure (MSAP), heart rate (HR) and total power density of vasomotor components (0–0.8 Hz) of systemic arterial pressure (SAP) spectrum in anaesthetized rats that received microinjection bilaterally into the rostral ventrolateral medulla (RVLM, at time 0) of artificial cerebrospinal fluid (aCSF), 17β-estradiol (E2β, 1 pmol), diarylpropionitrile (DPN, 1, 2 or 5 pmol) or 1,3,5-*tris*(4-hydroxyphenyl)-4-propyl-1 H-pyrazole (PPT, 5 pmol). Values are presented as the mean ± SEM; n = 6-8 animals per experimental group. ^*^P < 0.05 versus aCSF group in the post hoc Scheffé multiple-range test.

Compared with aCSF treatment, bilateral microinjection of E2β (1 pmol) into the functionally identified pressor region of RVLM resulted in significant decreases in MSAP and power density of vasomotor components of SAP spectrum, our experimental index for sympathetic neurogenic vasomotor outflow [17,22,23,27,30,32], with no apparent change in HR (Figure 1). Similar observations were found in animals that received microinjection of a selective ERβ agonist DPN (2 or 5 pmol) in the bilateral RVLM. The dose-dependent vasodepressive responses of DPN commenced approximately 30 min, and lasted for at least 120 min postinjection. At the low dose (2 pmol), DPN elicited vasodepressor effects that sustained for more than 3–4 hours postinjection, which similar duration was also observed at 1 pmol of E2β. Microinjection bilaterally into the RVLM of a selective ERα agonist PPT (5 pmol), on the other hand, did not affect basal hemodynamic parameters. Microinjection of the same doses of DPN (2 or 5 pmol) into the ventrolateral medullary areas adjacent to, but outside the confine of RVLM, e.g., spinal trigeminal nucleus or lateral paragigantocellular nucleus, also elicited minimal effects on the cardiovascular parameters (data not shown). Since DPN at 2 pmol produced moderate hypotension and reduction in sympathetic vasomotor tone, this dose was used in the subsequent experiments.

### Effects of ER antagonist on the ERβ agonist-induced vasodepressive responses

**Figure 2:**
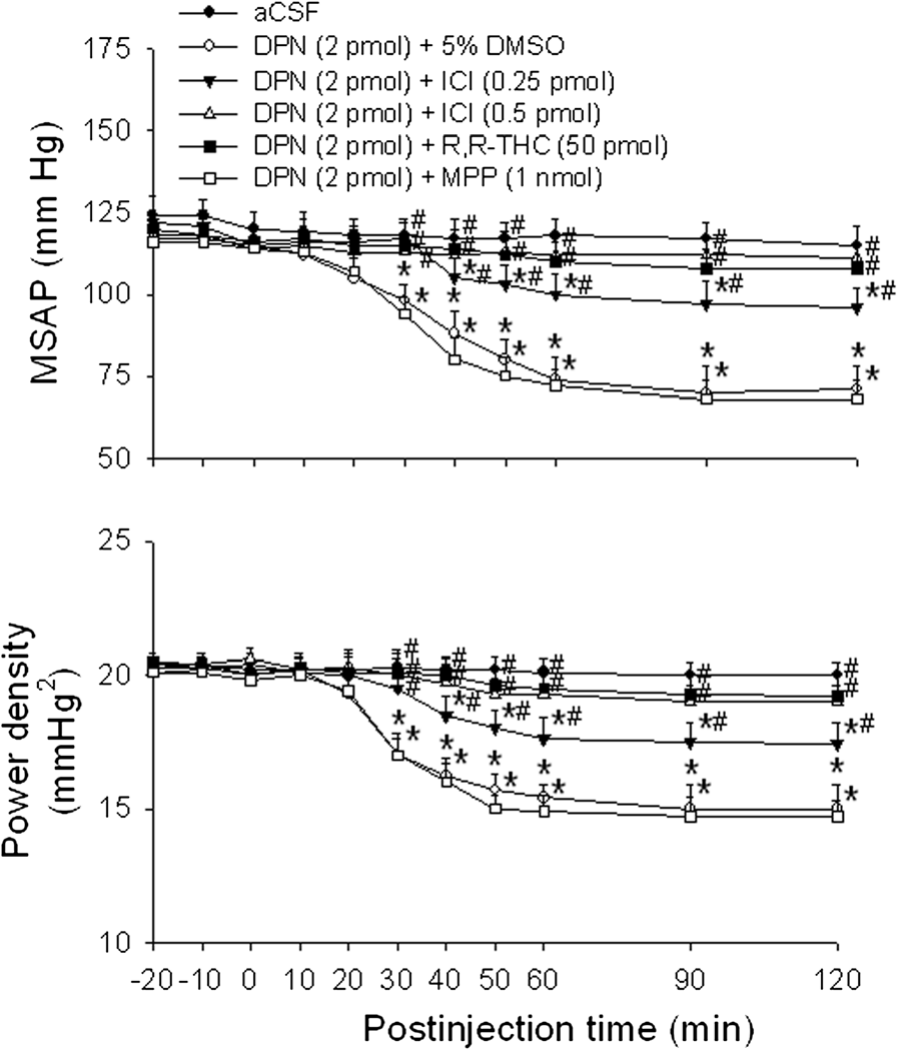
**Effects of ER antagonist on the E2β agonist-induced vasodepressive responses.** Time-course of the changes in MSAP and total power density of vasomotor components (0–0.8 Hz) of SAP spectrum in anaesthetized rats that received microinjection bilaterally into the RVLM (at time 0) of aCSF, or DPN (2 pmol) given together with ICI 182780 (ICI, 0.25 or 0.5 pmol), R,R-THC (50 pmol), MPP (1 nmol) or 5 % DMSO. Values are presented as the mean ± SEM; n = 6-8 animals per experimental group. ^*^P < 0.05 versus aCSF group and ^#^*P* < 0.05 versus DPN + DMSO group in the post hoc Scheffé multiple-range test.

Compared with aCSF or 5 % DMSO controls, co-administration bilaterally into the RVLM of a nonspecific ER antagonist, ICI 182780 (0.25 or 0.5 pmol) significantly attenuated the vasodepressive responses of DPN (2 pmol) in a dose-dependent manner (Figure 2). At the high dose (0.5 pmol), ICI 182780 almost completely blocked the DPN-induced hypotension and the decrease in sympathetic vasomotor tone. The vasodepressive responses induced by DPN (2 pmol) were also completely reversed by co-administration into the bilateral RVLM of a specific ERβ antagonist, R,R-THC (50 pmol), but not a specific ERα antagonist, MPP (1 nmol) (Figure 2). Complete attenuation was also obtained when R,R-THC (50 pmol) was microinjected into the bilateral RVLM at 20 min before DPN administration, whereas MPP (1 nmol) pretreatment was ineffective (data not shown). Microinjection bilaterally into the RVLM of ICI 182780 (0.25 or 0.5 pmol), R,R-THC (50 pmol) or MPP (1 nmol) alone, on the other hand, had no discernible effect on baseline MSAP, HR or power density of vasomotor components of SAP spectrum (Table 1).

**Table 1:** Effects of test agents on baseline MSAP, HR and power density of vasomotor components of SAP spectrum in RVLM

** *Treatment* **	** *Maximal changes in* **		
	***MSAP(mmHg)***	***HR(bpm)***	***Power Density(mmHg^2^)***
aCSF	+3.8 ± 0.5	+4.7 ± 0.9	+0.6 ± 0.7
ICI 182780 (0.25 pmol)	+4.3 ± 0.4	+5.2 ± 0.6	+0.6 ± 0.6
ICI 182780 (0.5 pmol)	+4.5 ± 0.5	+5.5 ± 0.3	+0.7 ± 0.5
R,R-THC (50 pmol)	+4.6 ± 0.7	+5.7 ± 0.8	+0.8 ± 0.7
MPP (1 nmol)	+5.3 ± 0.7	+5.1 ± 1.1	+0.8 ± 0.3
AMD (5 nmol)	−4.2 ± 0.6	+2.4 ± 1.2	−0.6 ± 0.7
AMD (10 nmol)	−4.5 ± 0.6	−3.4 ± 1.5	−0.6 ± 0.5
LY294002 (5 pmol )	+3.0 ± 0.7	−4.4 ± 1.0	+0.7 ± 0.6
Akt inhibitor (250 pmol)	+2.8 ± 0.8	+4.0 ± 0.8	+0.8 ± 0.4
AdGFP (5 × 10^8^ pfu)	−4.1 ± 0.8	+5.1 ± 1.2	−0.7 ± 0.6
AdPTEN (5 × 10^8^ pfu)	−4.8 ± 1.1	+5.7 ± 1.0	−1.0 ± 0.5
ERα ASON (250 pmol)	+5.5 ± 0.5	+5.5 ± 1.2	+0.6 ± 0.5
ERβ ASON (250 pmol)	+5.4 ± 1.0	+4.6 ± 1.1	+0.7 ± 0.7
ERα SCR (250 pmol)	+4.1 ± 0.8	+3.6 ± 1.0	+0.8 ± 0.7
ERβ SCR (250 pmol)	+4.9 ± 0.7	−3.9 ± 0.6	+0.7 ± 0.5

The baseline MSAP, HR and power density of vasomotor components of SAP spectrum were measured at 120 min, 24 h, or day 7 respectively, after microinjection bilaterally into RVLM of other test agents, ERα or ERβ ASON or SCR, or AdGFP or AdPTEN. All values are expressed as mean ± SEM; n = 6-7 animals per experimental group. Any significant differences (*P* < 0.05) were not detected between all the test agent groups and aCSF group in the Student’s *t*-test.

### Effects of ERα or ERβ ASON on the ERβ agonist-induced vasodepressive responses

**Figure 3:**
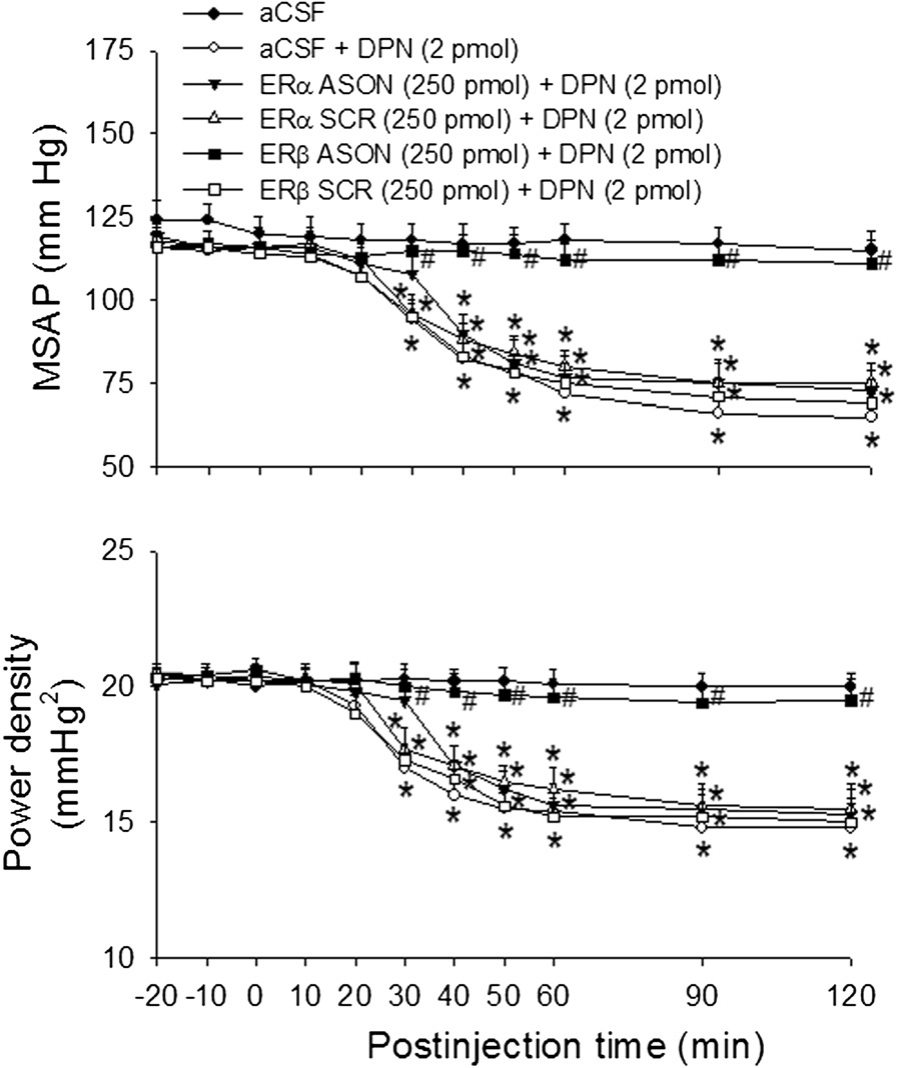
**Effects of ERα or ERβ ASON on the ERβ agonist-induced vasodepressive responses.** Time-course of the changes in MSAP and total power density of vasomotor components (0–0.8 Hz) of SAP spectrum in anaesthetized rats that received microinjection bilaterally into the RVLM (at time 0) of aCSF, or to rats pretreated with ERα ASON (250 pmol), ERα SCR (250 pmol), ERβ ASON (250 pmol), ERβ SCR (250 pmol) or aCSF, administered into the bilateral RVLM 24 hours before DPN (2 pmol) microinjection. Values are presented as the mean ± SEM; n = 6-7 animals per experimental group. ^*^P < 0.05 versus aCSF group and ^#^*P* < 0.05 versus aCSF + DPN group in the post hoc Scheffé multiple-range test.

Figure 3 shows that microinjection bilaterally of ERβ ASON (250 pmol) into the RVLM 24 hours before DPN (2 pmol) administration significantly attenuated the DPN-induced hypotension and reduction in power density of vasomotor components of SAP spectrum. Control application of ERα ASON, ERα SCR or ERβ SCR was ineffective. The ERα ASON (250 pmol), ERα SCR (250 pmol), ERβ ASON (250 pmol) or ERβ SCR (250 pmol), when administered bilaterally into the RVLM alone, did not affect the baseline MSAP, HR or power density of vasomotor components of SAP spectrum (Table 1).

### Effects of RNA synthesis inhibitor on the ERβ agonist-induced vasodepressive responses

**Figure 4:**
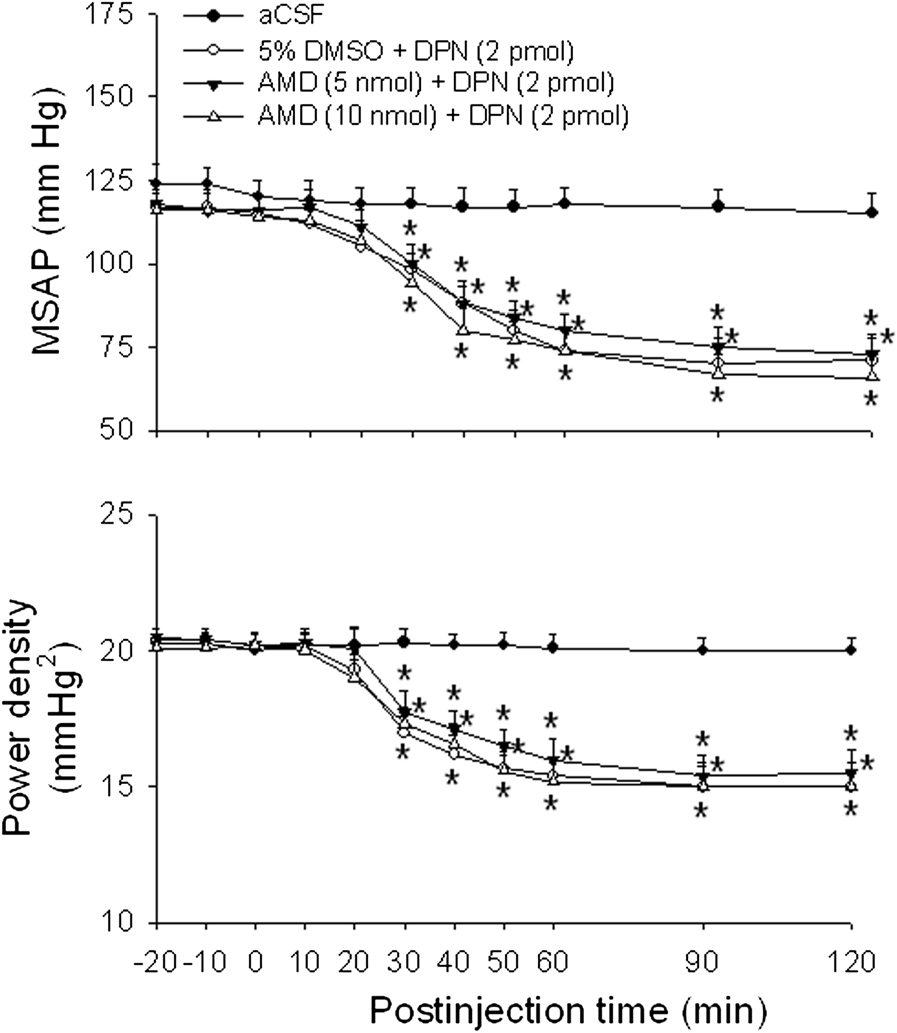
**Effects of RNA synthesis inhibitor on the ERβ agonist-induced vasodepressive responses.** Time-course of the changes in MSAP and total power density of vasomotor components (0–0.8 Hz) of SAP spectrum in anaesthetized rats that received microinjection bilaterally into the RVLM (at time 0) of aCSF, or to rats pretreated with actinomycin D (AMD, 5 or 10 nmol) or 5 % DMSO, administered into the bilateral RVLM 1 hour before DPN (2 pmol) microinjection. Values are presented as the mean ± SEM; n = 7-8 animals per experimental group. ^*^P < 0.05 versus aCSF group in the post hoc Scheffé multiple-range test.

We further investigate the involvement of nongenomic and/or genomic mechanisms in the DPN-induced cardiovascular depressive responses. Compared with 5 % DMSO controls, pretreatment with microinjection of a transcription inhibitor, AMD (5 or 10 nmol), into the bilateral RVLM 1 h prior to DPN administration (2 pmol), did not affect hypotension and reduction in power density of vasomotor components of SAP spectrum induced by DPN (Figure 4). Microinjection into bilateral RVLM of AMD (5 or 10 nmol) alone had no significant effect on baseline MSAP, HR or power density of vasomotor components of SAP spectrum (Table 1).

### Effects of PI3K inhibitor, Akt inhibitor or AdPTEN on the ERβ agonist-induced vasodepressive responses

**Figure 5:**
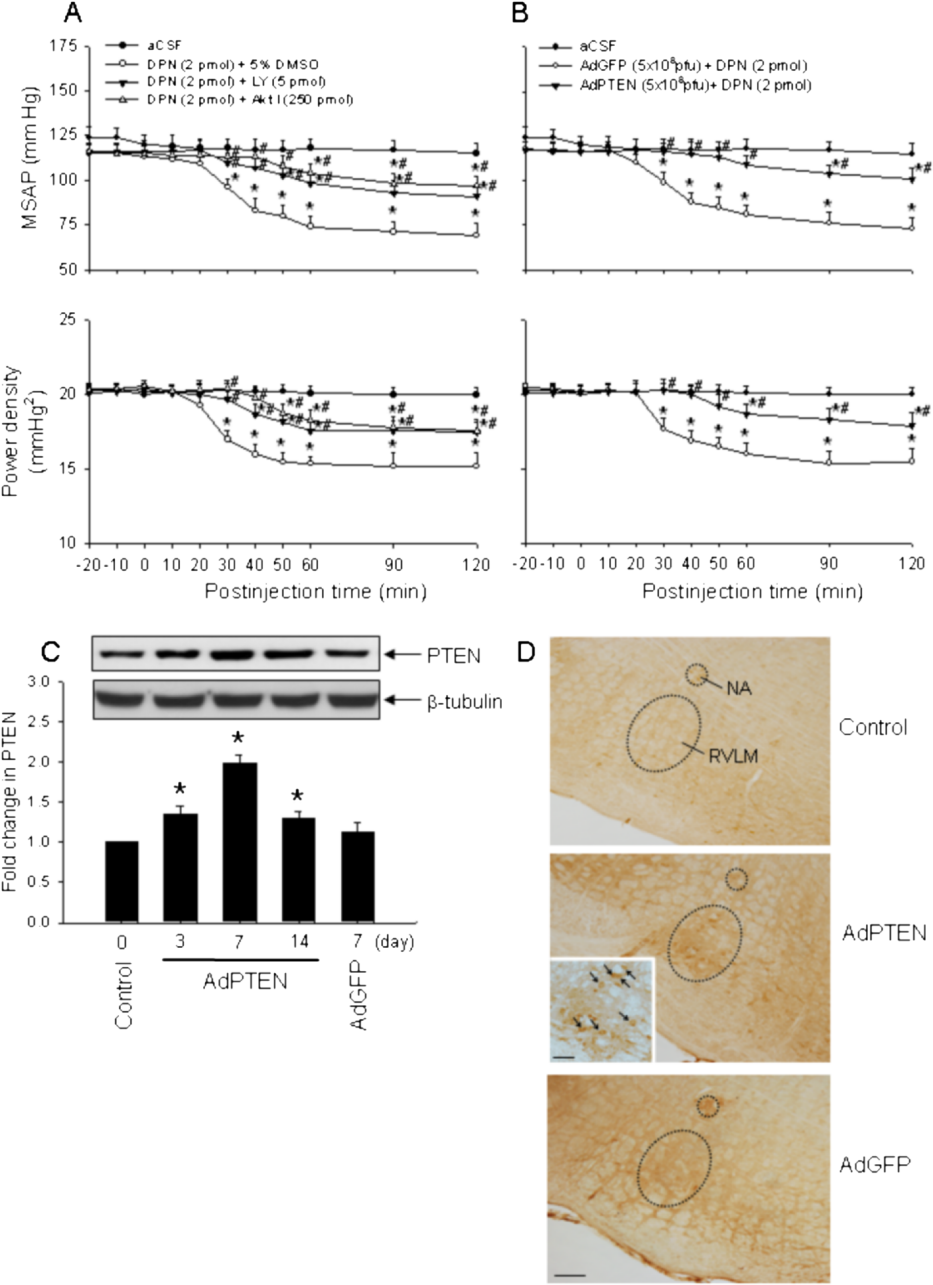
**Effects of PI3K inhibitor, Akt inhibitor or AdPTEN on the ERβ agonist-induced vasodepressive responses.** Time-course of the changes in MSAP and total power density of vasomotor components (0–0.8 Hz) of SAP spectrum in anaesthetized rats that received microinjection bilaterally into the RVLM (at time 0) of aCSF, or DPN (2 pmol) given together with LY294002 (LY, 5 pmol), Akt inhibitor (Akt I, 250 pmol) or 5 % DMSO (**A**), or with additional treatment with AdPTEN (5 × 10^8^ pfu) or AdGFP (5 × 10^8^ pfu), administered into the bilateral RVLM 7 days before DPN (2 pmol) microinjection (**B**). (**C**) Representative Western blots (insets) or densitometric analysis of the amount of PTEN protein relative to basal PTEN protein levels (control), detected from the RVLM 3, 7 or 14 days after animals received microinjection bilaterally into the RVLM of AdPTEN (5 × 10^8^ pfu) or AdGFP (5 × 10^8^ pfu). (**D**) Representative photomicrographs showing the distribution of PTEN-immunoreactivity (arrows) in RVLM on 7 days after animals received bilateral microinjection into the RVLM of aCSF (control), AdPTEN (5 × 10^8^ pfu) or AdGFP (5 × 10^8^ pfu). Calibration bar: 100 μm in low magnification and 50 μm in high magnification of inset. Values are presented as the mean ± SEM; n = 6-7 animals per experimental group. ^*^P < 0.05 versus aCSF or control group and ^#^*P* < 0.05 versus DPN + DMSO or AdGFP + DPN group in the post hoc Scheffé multiple-range test. NA, nucleus ambiguus; RVLM, rostral ventrolateral medulla.

To decipher the role of PI3K/Akt signaling pathway in the DPN-induced vasodepressive responses, we evaluated the effects of PI3K inhibitor, Akt inhibitor or AdPTEN in the RVLM on vasodepressive responses promoted by DPN. In contrast to vehicle injection, co-administration bilaterally into the RVLM of a PI3K inhibitor, LY294002 (5 pmol), or an Akt inhibitor (250 pmol), significantly decreased the vasodepressive responses of DPN (2 pmol) (Figure 5A). Similar results were obtained when LY294002 or Akt inhibitor was administered into the bilateral RVLM 30 min before DPN microinjection (data not shown). We also found that DPN-induced hypotension and reduction in power density of vasomotor components of SAP spectrum were significantly reversed 7 days after the gene transfection of AdPTEN (a total of 5 × 10^8^ pfu) to the bilateral RVLM (Figure 5B). Microinjection bilaterally into the RVLM of AdPTEN resulted in a significant increase in PTEN protein expression, which peaked on day 7 after the gene transfer (Figure 5C). On day 7 after the gene transfection, distribution of PTEN-positive immunoreactivity was also significantly increased in RVLM (Figure 5D). Microinjection bilaterally into the RVLM of LY294002 (5 pmol), Akt inhibitor (250 pmol), AdGFP (5 × 10^8^ pfu) or AdPTEN (5 × 10^8^ pfu) alone had no effect on baseline MSAP, HR or power density of vasomotor components of SAP spectrum (Table 1).

### Microinjection sites

**Figure 6:**
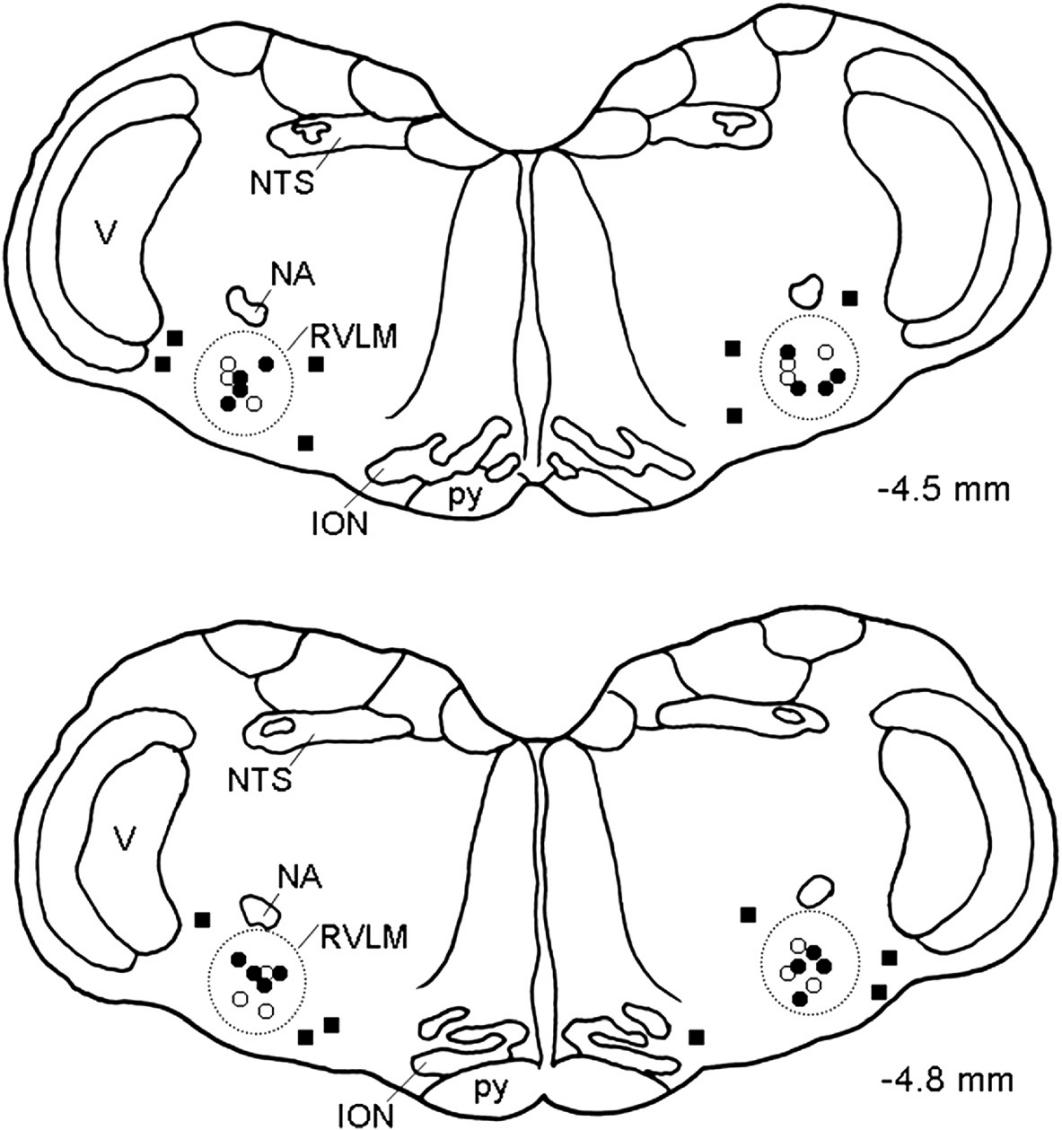
**Microinjection sites of E2β and DPN in the RVLM.** Diagrammatic representations of two rostral-caudal levels of the RVLM (dotted line areas) with reference to the lambda illustrating the location of sites where bilateral microinjection of E2β (○, 1 pmol) and DPN (●, 2 pmol) elicited significant inhibitory or minimal effects (■, non-RVLM microinjection sites) on the MSAP and power density of vasomotor components of SAP spectrum. Numbers on right side indicate distance from the lambda. For the purpose of clarity, approximately 10 % of the total microinjection sites are included and are presented on both side of the diagram. ION, inferior olivary nucleus; NA, nucleus ambiguous; NTS, nucleus tractus solitarii; RVLM, rostral ventrolateral medulla; V, nucleus of the spinal trigeminal nerve; py, pyramidal tract.

Histological verification of locations of micropipette tips in the ventrolateral medulla confirmed that all observations were made from animals that received local administration of the test agents within the anatomic confines of the RVLM (Figure 6). For the purpose of clarity, Figure 6 only summarizes the location of sites where microinjection of E2β (1 pmol) or DPN (2 pmol) exhibited significant (*P* < 0.05) inhibitory effects on the MSAP and power density of vasomotor components of SAP spectrum.

## Discussion

The major finding of the present study is that nontranscriptional activation of PI3K/Akt signaling mediates the short-term hypotension and reduction in sympathetic vasomotor tone induced by ERβ activation in the RVLM. It is well established that the sympathetic premotor neurons within the RVLM plays a pivotal role in the generation and maintenance of the tonic sympathetic vasomotor outflow [21]. It is also known that spectral vasomotor components (very low-frequency and low-frequency) of SAP signals take origin from these RVLM neurons [25]. In agreement with our recent observations [17], we found in the present study that direct microinjection of E2β into the RVLM of male rats elicited significant decreases in SAP and power density of the vasomotor components of SAP signals, our experimental index for sympathetic neurogenic vasomotor outflow [17,22,23,27,30,32]. In addition, we found closely correlated temporal profiles between the reduced power density of the vasomotor components of SAP signals and hypotensive effect after microinjection of E2β or ERβ agonist into the bilateral RVLM, suggesting that estrogen can acting directly on the RVLM neurons to reduce the tonic sympathetic vasomotor outflow, resulting in a decrease of SAP. In the present study, we purposely used male animals to avoid confounding cardiovascular effects induced by differing levels of circulating estrogen in female rats. Subramanian and colleagues [34] recently demonstrated in female rats that chronic exposure to low levels of E2β by peripheral slow-release pellets caused significant increases in superoxide production in the RVLM and SAP. Whether the differential cardiovascular responses to E2β activation is gender dependent or may result from the different amounts of estrogen distributed in the RVLM caused by different routes of E2β administration, however, await further delineation.

Both ERα and ERβ are distributed in the RVLM neurons [20,28]. Similar to our previous report [17], at the receptor level, we found that activation of ERβ in the RVLM contributes predominantly to the vasodepressor effect of estrogen. E2β and the ERβ-selective agonist DPN, but not the ERα-selective agonist PPT, induced vasodepressive effects that were almost completely antagonized by the nonselective ER antagonist, ICI 182780, or the ERβ-selective antagonist, R,R-THC. We also found that blockade of ERβ mRNA expression with a bilateral microinjection into the RVLM of the ERβ ASON significantly attenuated the vasodepressive effects induced by DPN. ERα ASON treatment, on the other hand, did not cause a significant change in DPN-induced vasodepressive effects. Together, these findings suggest that ERβ plays a predominant role in mediating the vasodepressive effects of estrogen in the RVLM. In support of this suggestion, estrogen-induced rapid inhibition of voltage-gated Ca^++^ currents was mediated predominantly by ERβ in whole-cell patch clamp recordings from the isolated RVLM neurons [28]. Moreover, the phenotypes observed for ERβ knockout mice confirm that ERβ is necessary for the regulation of vascular function and blood pressure [35]. Examined under electron microscopic level, ERβ-immunoreactivity in the RVLM neurons is more commonly found in somata and dendrites, particularly on plasma membranes, endomembranes and mitochondria [28], raising therefore a possibility that these primarily extranuclear ERβ in the RVLM are involved in the rapid, nongenomic vasodepressor effects of estrogen. In accordance with these cellular observations, we found that both E2β and ERβ agonist caused rapid and short-term vasodepressor responses that occurred approximately 30 min and sustained for more than 3–4 hours postinjection. A lack of effect on the observed short-term vasodepressive responses by AMD further indicates this process does not require the classical nuclear effects of estrogen [20,28]. Moreover, these short-term responses were almost completely inhibited by co-microinjection with the ER antagonist, ICI 182780, or the ERβ-selective antagonist, R,R-THC, suggesting that E2β-dependent short-term vasodepressor responses may be mediated via the rapid nongenomic activation of ERβ. Minimal alterations in basal SAP, HR or sympathetic neurogenic vasomotor tone following application of ERβ antagonist or ERβ ASON into the RVLM suggest that under physiologic circumstances endogenous estrogen and ERβ activation at the RVLM are not critical for tonic regulation of cardiovascular performance in male animals. It is noteworthy that these cardiovascular regulatory effects of the female steroids are site specific, since application of the same agents to areas outside the confines of the RVLM where ERs are sparse or not existed [19,20], did not significantly influence the baseline hemodynamic parameters.

Another important contribution of this study is the establishment of an intracellular signaling mechanism of ERβ-dependent nongenomic cardiovascular actions by estrogen in the RVLM. Our study provided novel evidence to suggest that the vasodepressive effects after nontranscriptional activation of ERβ in the RVLM may involve activation of the intracellular PI3K/Akt signaling pathway. Recently, rapid, nontranscriptional mechanisms of signal transduction through activated ERs have been identified. In response to estrogen, ERs can interact with the p85 regulatory subunit of PI3K and activates the PI3K/Akt signaling pathway [5,36]. Furthermore, the PI3K/Akt signaling pathway has been indicated to play a crucial role in the cardiovascular protective effects of estrogen [5-7]. In the present study, the specific PI3K inhibitor or Akt inhibitor effectively decreased the vasodepressive effects induced by ERβ activation. However, because application of LY294002 or Akt inhibitor did not completely abolish the ERβ-mediated cardiovascular responses, alternative signaling pathways such as extracellular signal-regulated kinase/mitogen-activated protein kinases, tyrosine kinases or transmembrane G-protein-coupled receptor GPR30 [5,37] in mediating the ERβ-dependent nongenomic cardiovascular actions cannot be ruled out.

It is well established that PTEN is a dual phosphatase that was originally identified as a tumor suppressor protein and plays a major negative regulator in the PI3K/Akt signaling pathway [38,39]. Therefore, we overexpressed the PTEN transgene by the recombinant AdPTEN to downregulate PI3K activity in the RVLM. In consistent to the findings reported previously [40], AdPTEN transfection increased PTEN expression and the distribution of PTEN-positive cells in the RVLM. The same treatment also resulted in a significant reversal of the ERβ-mediated vasodepressive effects similar to that evoked by PI3K and Akt inhibitors. Together, these observations strongly support the PI3K/Akt signaling pathway in the E2β-induced vasodepressive effects in the RVLM. We reported previously that nitric oxide (NO) participates in the ERβ-mediated vasodepressive responses in the RVLM [17]. Whether there is a nontranscriptional crosstalk between ERβ, PI3K/Akt signaling pathway and NO production in the RVLM to contribute to central regulation of cardiovascular functions, however, remains to be clarified. Moreover, the renin-angiotensin system in RVLM also plays an active role in central regulation of cardiovascular function and pathogenesis of hypertension [41]. In view that estrogen levels can selectively affect the expression and subcellular distribution of the angiotensin type 1 receptors within RVLM neurons [42], and that PI3K/Akt signaling pathways is involved in angiotensin 1-7-induced protection against cardiac hypertrophy [43], interactions between ERs and renin-angiotensin system at RVLM in central cardiovascular regulation also warrant further investigation.

## Conclusions

In conclusion, our results demonstrate for the first time that the ERβ-mediated hypotension and reduction in sympathetic vasomotor tone are dependent on the nontranscriptional activation of intracellular PI3K/Akt signaling pathway in the RVLM. This finding further expands the current mechanistic views on estrogen-replaced cardiovascular protection in postmenopausal females. In view of a growing incidence of hypertension in the postmenopausal female, elucidation of these intracellular signaling cascades may therefore provide new information to link estrogen deficiency with hypertension and other cardiovascular diseases.

## Abbreviations

aCSF, Artificial cerebrospinal fluid; AdGFP, Adenovirus encoding GFP; AdPTEN, Adenovirus encoding PTEN; Akt, Serine/threonine kinase Akt; AMD, Actinomycin D; ANOVA, Analysis of variance; ASON, Antisense oligonucleotide; DMSO, Dimethyl sulfoxide; DPN, Diarylpropionitrile; E2β, 17β-estradiol; ER, Estrogen receptor; GFP, Green fluorescence protein; HR, Heart rate; MPP, Methyl-piperidino-pyrazole; MSAP, Mean SAP; NO, Nitric oxide; pfu, Plaque-forming units; PI3K, Phosphatidylinositol 3-kinase; PPT, 1,3,5-tris(4-hydroxyphenyl)-4-propyl-1 H-pyrazole; PTEN, Phosphatase and tensin homologues deleted on chromosome 10; R,R-THC, R,R-tetrahydrochrysene; RVLM, Rostral ventrolateral medulla; SAP, Systemic arterial pressure; SCR, Scrambled.

## Competing interests

The authors declare that they have no competing interests.

## Authors’ contributions

KLHW and CHC carried out the neuropharmacological and biochemical experiments, and performed the Western blotting analysis. CDS participated in experimental conception and design, performed animal and immunohistochemical experiments, acquisition of data, the statistical analysis and interpretation of data, and drafted and revised the manuscript. All authors read and approved the final manuscript.
